# Recent progress of covalent organic frameworks membranes: Design, synthesis, and application in water treatment

**DOI:** 10.1016/j.eehl.2023.07.001

**Published:** 2023-07-13

**Authors:** Xiaolu Liu, Yang Li, Zhongshan Chen, Hui Yang, Suhua Wang, Zhenwu Tang, Xiangke Wang

**Affiliations:** a MOE Key Laboratory of Resources and Environmental System Optimization, College of Environmental Science and Engineering, North China Electric Power University, Beijing 102206, China; b School of Environmental Science and Engineering, Guangdong University of Petrochemical Technology, Maoming 525000, China; c College of Life and Environmental Sciences, Minzu University of China, Beijing 100081, China

**Keywords:** Covalent organic frameworks, Covalent organic frameworks membranes, Preparation strategies, Characterization techniques, Water treatment

## Abstract

To date, significant efforts have been devoted to eliminating hazardous components to purify wastewater through the development of various nanomaterials. Covalent organic frameworks (COFs), an important branch of the porous crystalline family, possess the peculiarity of ultrahigh surface area, adjustable pore size, and facile functionality. Exciting studies from design fabrication to potential applications in water treatment by COF-based membranes (COMs) have emerged. This review summarizes various preparation strategies and synthesis mechanisms for COMs, including layer-by-layer stacking, *in situ* growth, interfacial polymerization, and electrochemical synthesis, and briefly describes the advanced characterization techniques for COMs. Moreover, the application of COMs in heavy metal removal, dye separation, purification of radionuclides, pollutant detection, sea water desalination, and so on, is described and discussed. Finally, the perspectives on future opportunities for designing COMs in water purification have been proposed.

## Highlights

• Analyzing the advantages of covalent organic frameworks for the preparation of COF-based membranes (COMs).
• Advanced characterization techniques for COMs are described.
• Various strategies for preparation of COMs are comprehensively summarized.
• Summarizing the application of COMs in water treatment and analyzing the potential mechanism.

## Graphical abstract

**Figure:**
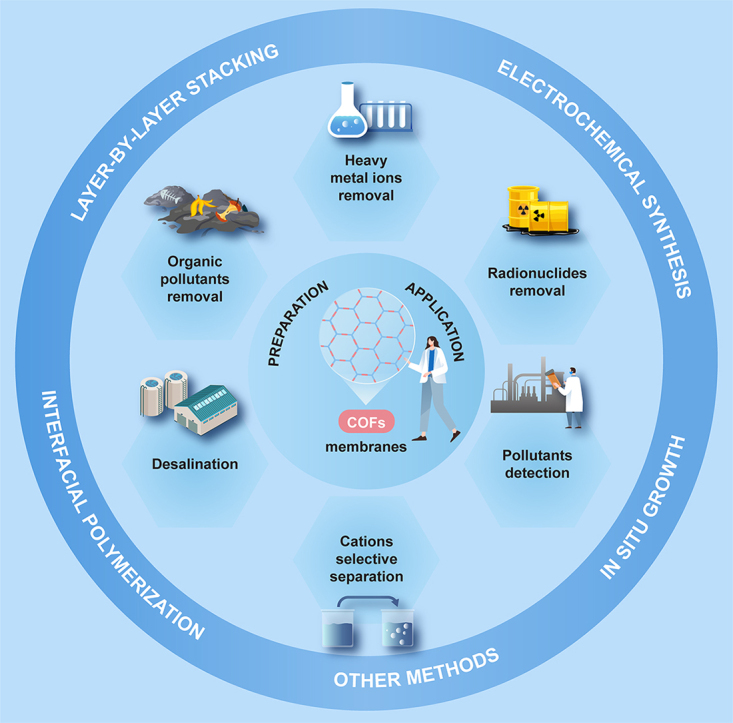

## 1 Introduction

Water, as a precious renewable resource, is significant to all creatures on the earth [1,2]. However, the increasing water pollution led by industrial emissions, agricultural activity, and municipal domestic sewage seriously threatens the global water security [[3], [4], [5], [6], [7], [8], [9]]. Most pollutants in polluted water are poisonous and difficult to degrade, thereby posing a significant long-term risk to humans [10]. In addition, with the development of the world economy, the growing population, rapid urbanization, and worldwide freshwater resources will face a serious shortage crisis [11]. To ensure the safety and sufficiency of water resources, various methods and advanced nanomaterials have been developed to eliminate contaminants from the water environment. However, a crucial challenge to conquer is how to efficiently purify polluted water.

Various techniques, including adsorption [12,13], ion exchange [14,15], electrochemistry [16,17], and photocatalysis [18], have been used for sewage purification. However, traditional nanomaterials, such as clays, layered double hydroxides, and mesoporous carbon, exhibit poor decontamination capabilities and limited selectivity [11]. Activated carbon and organic polymer always exhibit slow adsorption kinetics due to their small pore volumes or pore sizes [19,20]. Furthermore, some materials, such as organic resins, layered metal sulfides, and so on, possess poor recycling and reusable performance or relatively chemical and thermal stability, which possibly lead to secondary pollution of the water environment, and do not meet the principles of green, energy-saving, and renewable of modern environmental protection [21]. Thereby, developing an efficient and economical nanomaterial with high specific surface area, enhanced pollutant removal capabilities, and approving reusability is desperately desired. Recently, advanced porous materials such as covalent organic polymers (COPs) [8], metal-organic frameworks (MOFs) [22,23], porous organic polymers ​[24,25], hydrogen-bonded organic frameworks (HOFs) [26], and covalent organic frameworks (COFs) [27], afford broad platform to design nanomaterials with high efficiencies to segregate contaminants from water.

As an important branch of the crystalline porous materials family, COFs constructed by organic linkers *via* reversible covalent bonds possess periodically extended topology network structures [28]. The unique structure endows COFs with adjustable pore size and structure, super surface area, low density, and facilely tailored functionality, arousing extensive interest in electrocatalysis, photocatalysis, adsorption, and membrane separations [29,30]. Meanwhile, these unparalleled advantages make COFs promising candidates for fabricating advanced membrane materials. Compared to powdered COFs, COF-based membranes (COMs) possess many advantages in the field of separation [31]. First, the precisely defined pore sizes of the COMs can be designed by selecting ligands through a preselected design to separate guest molecules with different van der Waals volumes [32]. Moreover, functional COMs could be precisely adjusted by grafting various functional groups on the organic linker, thus highly controlling the host-guest interaction in an attractive or repulsive manner. Furthermore, COMs afford unique superiority in segregating contaminants from water, which avoids the challenges of powder material recycling and collection difficulties.

Exciting studies focusing on synthetic strategies and separation applications of COMs have appeared. Although excellent research about applications in water treatment, including wastewater purification and desalination by COMs, has been reported, there is no accessible, comprehensive review of COMs that focuses specifically on this area to date. For example, Yuan et al. discussed the applications of COMs in gas separation, water treatment, organic solvent nanofiltration, pervaporation, and fuel cells [32]. Wang et al. summarized the advancements of COFs in gas phase separation and liquid phase separation [33]. Therefore, we first give the latest survey on water treatment applications by COMs. This review summarizes the latest research achievements for COMs synthesis strategy, including layer-by-layer stacking, *in situ* growth, interfacial polymerization (IP), and electrochemical synthesis, and the preparation mechanism is discussed in detail. Furthermore, the applications of COMs in water treatment, including heavy metal removal, dye separation, purification of radionuclides, pollutant detection, seawater desalination, and so on, are summarized. In addition, the advanced characterization techniques for COMs are also briefly described. The peculiarity and design principles of COFs in water treatment are also highlighted. Finally, some observations are presented on the opportunities and challenges for the further development of COMs in design synthesis and water treatment.

## 2 Synthesis strategies for COMs

**Fig. 1:**
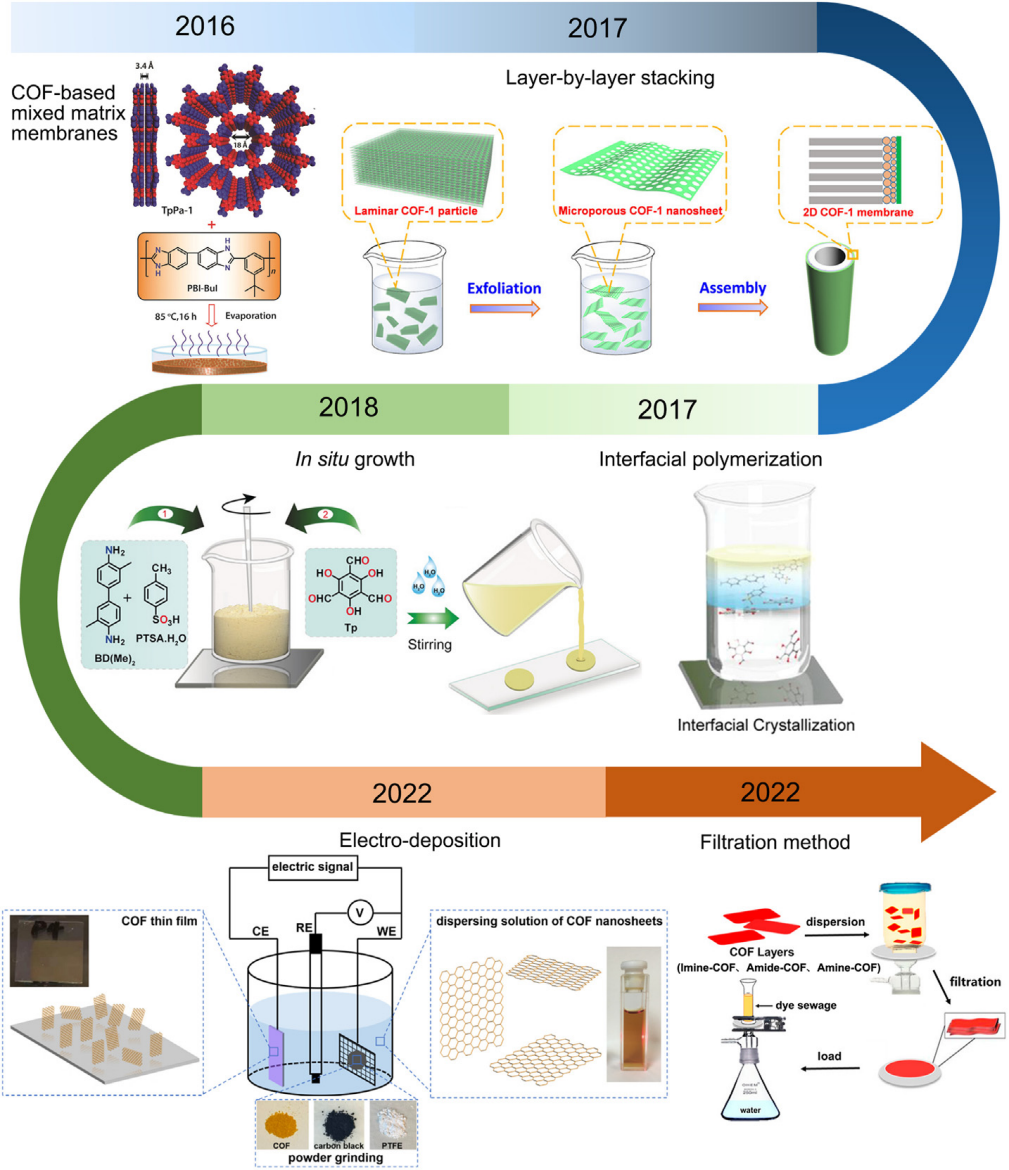
Developmental milestones of COMs preparation method [[35], [36], [37], [38], [39],^53^]. Panels reprinted with permission from: COF-based mixed matrix membranes, ref. [35], John Wiley and Sons; Layer-by-layer stacking, ref. [36], American Chemical Society; Interfacial polymerization, ref. [53], American Chemical Society; *In situ* growth, ref. [39], John Wiley and Sons; Electro-deposition, ref. [37], American Chemical Society; Filtration method, ref. [38], American Chemical Society. COF, covalent organic framework; COM, COF-based membrane.

The special advantage of orderly arranged structure, regular pore size, and high pore density endows COFs with unique advantages in membrane applications compared with other porous nanomaterials. In the initial stage of preparing the COMs, COFs are usually mixed with organic polymers to prepare COF-based mixed matrix membranes (MMMs). However, this method is difficult to synthesize continuous COMs with high selectivity [34]. With the development of membrane preparation and COFs synthesis technologies, such as layer-by-layer stacking, IP, *in situ* growth, electro-deposition, filtration method, and so on, have been applied. Fig. 1 shows the various methods and their synthesis diagram for the preparation of COMs. Biswal et al. prepared COF and polybenzimidazole ​composite membranes with high chemical stability and flexibility in 2016, which showed an exciting performance in gas separation [35]. Li et al. reported a novel COF-1 membrane *via* layer-by-layer stacking in 2017 [36]. With the increasing development of COMs preparation technology, *in situ* growth, electro-deposition, and filtration method have been explored for the preparation of COMs [[37], [38], [39]]. The preparation process, mechanism, and characteristics of each method will be discussed in detail in this section.

### 2.1 Layer-by-layer stacking

**Fig. 2:**
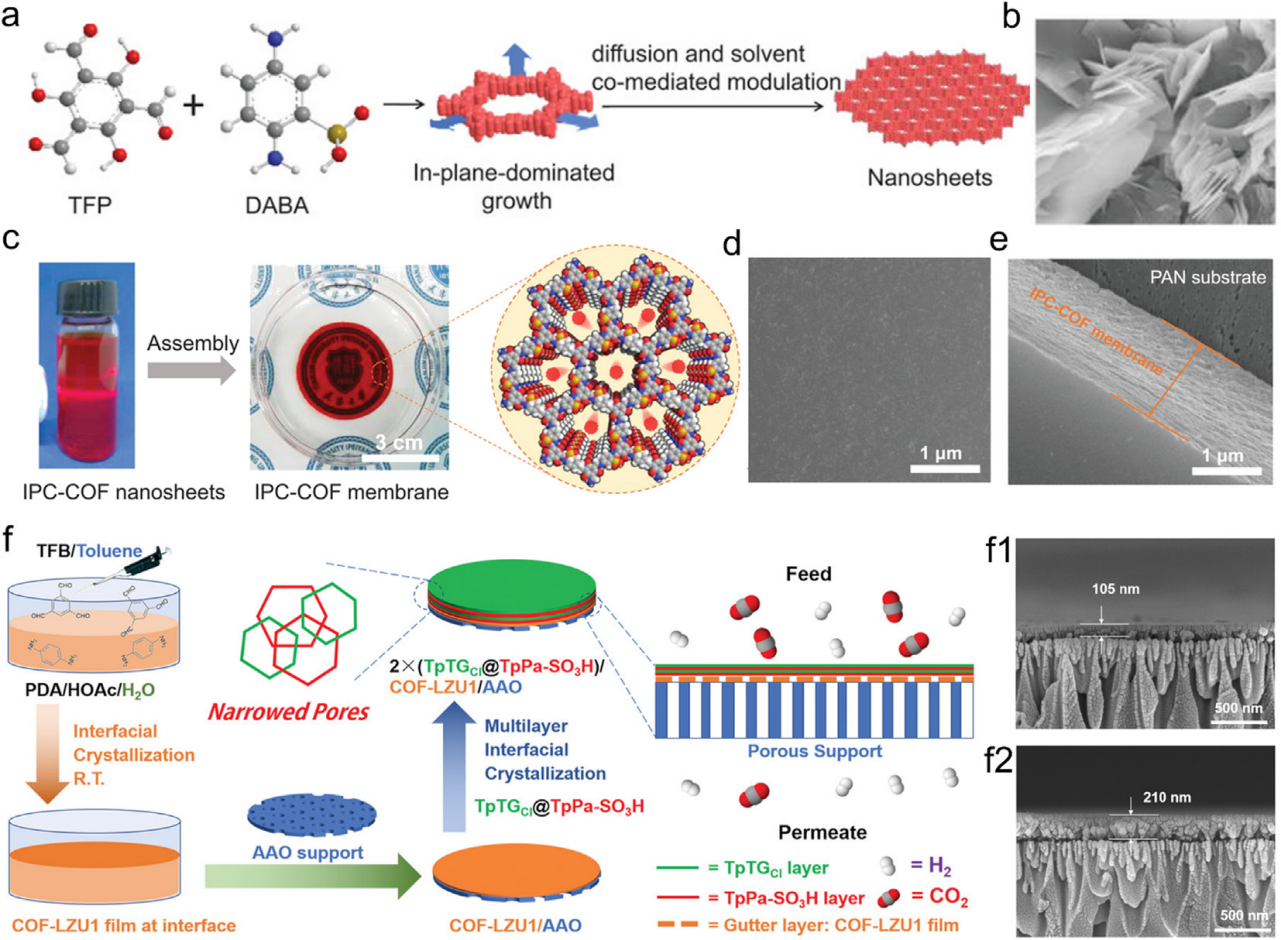
COMs preparation by layer-by-layer stacking strategy. (**a**) Schematic illustration of COF nanosheets; (**b**) SEM images of COF nanosheets; (**c**) schematic illustration of COMs assembly and pore structures; SEM images of COMs (**d**) surface and (**e**) cross-section [40]; (**f**) the fabrication of COMs COF-LZU1 with narrowed apertures using a multi-interfacial engineering strategy, and (f1, f2) SEM images of multilayer COMs with different thickness [42]. Panels reprinted with permission from: a–e, ref. [40], John Wiley and Sons; f, ref. [42], John Wiley and Sons. SEM, scanning electron microscopy.

The fabrication of COMs by stacking layers generally requires two processes. First, the COFs need to be dispersed in water or solvent to form bulk nanosheets. Most COFs have a regular 2D layered structure; by breaking the van der Waals forces between adjacent layers, 2D COF nanosheets can be easily obtained. Then, the obtained COF nanosheets are superimposed on a porous substrate by pressure or vacuum-assisted filtration to generate continuous ultrathin COMs. By changing the number of COF nanolayers, the thickness of thin COMs can be controlled. The thin COMs prepared by this method have continuous 1D nanopore channels, showing high electron and mass transport rates. For example, Cao et al. prepared COF nanosheets with sulfonic acid groups in water solutions *via* ​the diffusion and solvent co-mediated modulation (Fig. 2a) [40]. The slow diffusion of monomer in solvent and the interaction between COFs and solvent lead to the growth of the nucleation plane, forming COF nanosheets. The scanning electron microscopy (SEM) images in Fig. 2b clearly show nanosheet’ structures. The COF nanosheets were assembled by filtration to obtain robust COMs (IPC-COF membranes) (Fig. 2c). The SEM images indicated the highly uniform surface and cross-section of IPC-COF membranes (Figs. 2d and e). The thickness of IPC-COF membranes was tuned by varying the volume of IPC-COF nanosheet suspensions. With membrane electrode assemblies (MEAs) in proton exchange membrane fuel cell (PEMFC) application, the MEA-IPC-COF presented weakly humidity-dependent conductivity, high peak power density, and unprecedentedly high proton conductivity. Similarly, Li et al. fabricated an ultrathin COMs with excellent thermal stability and highly permeable performance by dipping and coating COF nanosheets on the carrier multiple times and then drying them at room temperature [36]. Recently, Wang et al. intelligently utilized the adhesion of COF nanosheets (TpPa-SO_3_H) and TpTTPA nanoribbons to synthesize COMs [41]. The electrostatic and π–π interactions between TpPa-SO_3_H and TpTTPA nanoribbons induce COF nanosheets to form ordered and robust COMs *via* vacuum-assisted self-assembly. The continuous and independent COF layer of about 630 ​nm was clearly observed by SEM images. For comparison, COF nanosheets without TpTTPA nanoribbons would form fragmentary COMs.

An interesting work of Ying et al. proposed a multi-interfacial engineering strategy and successfully prepared COMs *via* a layer-by-layer stacking method [42]. Specifically, they used two COFs (TpPa-SO_3_H and TpTGCl) with different aperture sizes to form narrowed apertures at the COF-COF interfaces *via* direct layer-by-layer growth manner atop another large-pore COF (COF-LZU1) film gutter layer (Fig. 2f). The thicknesses of COF-LZU1 film could be controlled between 105 and 210 ​nm by growing different molecular layers (Figs. 2f1 and 2f2). The prepared COF-LZU1 membranes by multi-interfacial engineering strategy possess more COF-COF interfaces and thinner thicknesses, resulting in high sieving capability and reducing the transport resistance in the field of gas separation. More importantly, this study has implications for the interface and pore engineering operations of other COFs and porous materials and their membranes. In general, the COMs prepared by the layer-by-layer stacking method are thin, and the preparation conditions are demanding, and not suitable for large-scale synthesis.

### 2.2 *In situ* growth

*In situ* growth, another important method to prepare COMs, was pioneered in 2017 by Banerjee et al. [43]. Generally speaking, precursor solution containing organic ligands was first prepared, and then the suitable substrate was selected and charged with the precursor solution. Subsequently, the substrate was positioned into the reaction vessel and heated at a set temperature for a fixed reaction time. Next, the substrate was thoroughly washed with organic solvents or deionized water to remove the reaction mixture to obtain homogeneous COMs on the substrate. The thickness of COMs is related to baking time, spin-coating cycles, and other factors, and the uniformity and quality of the COMs are greatly affected by the substrate. In the preparation of the COMs process, *p*-toluene sulfonic acid (PTSA), water, or small acidic molecules could be added to serve as co-reagent during the baking process to promote the crystallinity as well as increase the porosity of the COMs [39,43]. COMs possess many advantages in target applications. For example, when serving as MEA membranes, the entrapment of this efficient proton carrier of PTSA allowed excellent proton-conducting ability in the COMs, resulting in a significant promotion in proton conductivity [39]. The common substrate used to prepare the COMs included teflon substrate [44], glass plate [43], silicon substrate, stainless-steel net (SSN) substrate, fluorine-doped tin oxide (FTO) [45], aluminum oxide, graphene oxide [46], and so on. Tailored COMs could also be grown directly on conducting substrates and subsequently serve as electrodes in device applications and others [47].

**Fig. 3:**
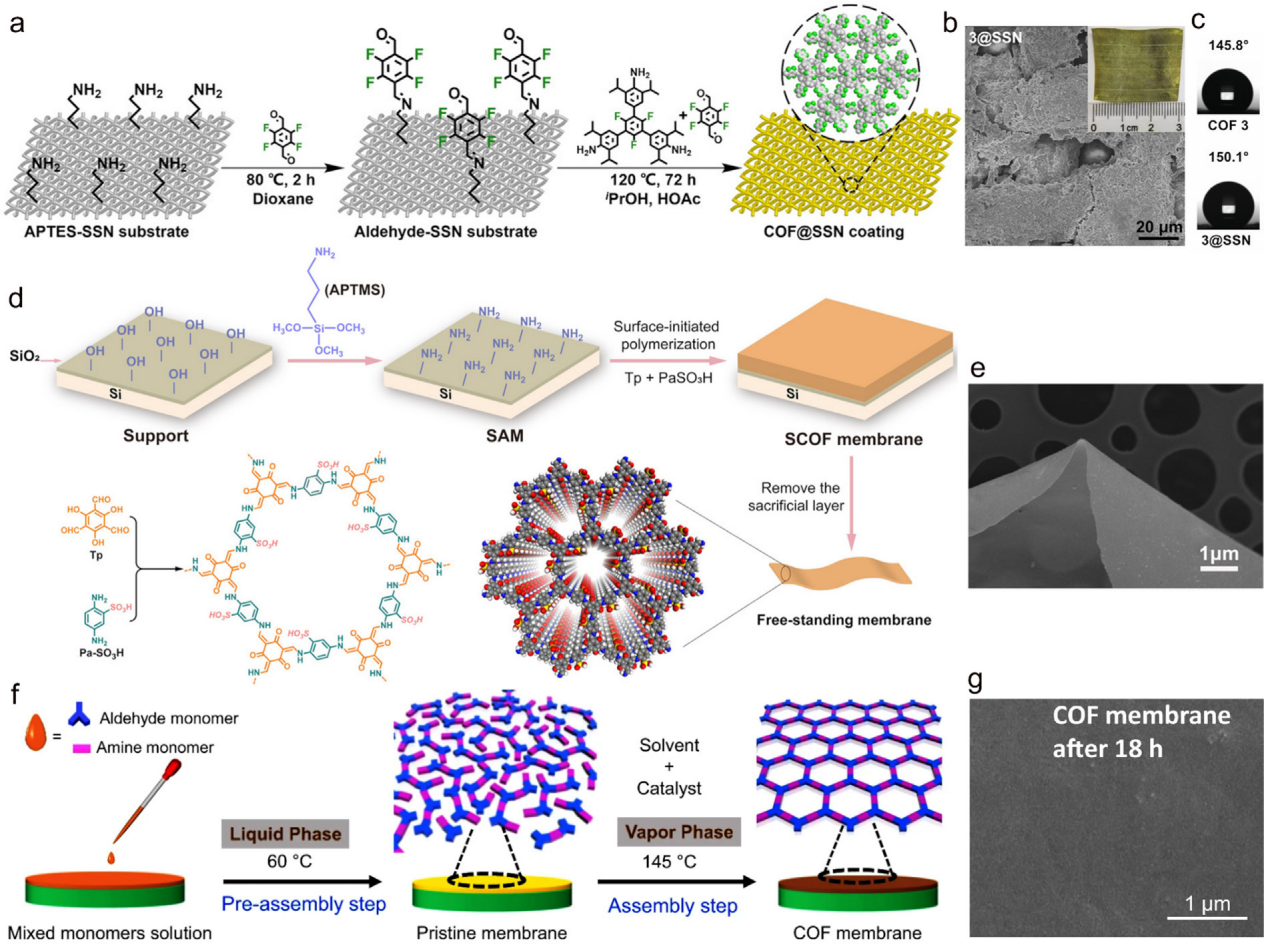
COMs preparation by *in situ* growth strategy. (**a**) Schematic synthesis of COF coating on modified SSN substrate; (**b**) SEM image of 3@SSN coating; (**c**) the water contact angle of 3@SSN [49]; (**d**) preparation of the SCOF membrane grafted on silicon wafers and the free-standing SCOF membrane; (**e**) SEM image of the free-standing SCOF [50]; (**f**) free-standing COMs grow on ITO substrate and (**g**) the surface SEM image of COMs [52]. Panels reprinted with permission from: a–c, ref. [49], John Wiley and Sons; d–e, ref. [50], John Wiley and Sons; f–g, ref. [52], Springer Nature. ITO, indium tin oxide; SCOF, sulfonic COF; SSN, stainless-steel net.

Based on goal application, *in-situ* growth of COMs directly on electrodes or conductive substrates has been reported. For example, Bein et al. directly integrated the oriented thin COMs into a device as model systems to study their electrical properties [48]. They also reported a study that took an imine-based COF to be *in-situ* grown as an oriented film on conducting substrates, then as photoelectrodes for photocatalytic hydrogen evolution [47]. The highly oriented COMs absorbed light in the visible range and produced photoelectrons that diffused to the surface and were transferred to the electrolyte, leading to a hydrogen evolution reaction. Experiments demonstrated that the photocurrent obtained by loading Pt nanoparticles on the COMs photoelectrode is increased by four times. Due to the uniform pore size, enhanced chemical stability, and excellent mechanical robustness, Liu et al. prepared COMs with controllable hydrophobicity and processability through directly growing them on SSN substrate with an average pore diameter of about 30 ​mm by *in-situ* growth strategy and applied them to oil–water separation [49]. Fig. 3a exhibits the schematic preparation synthesis of COMs coating on a 3-aminopropyltriethoxysilane-modified SSN substrate. The SEM images of as prepared COMs (3@SSN) are shown in Fig. 3b. We can clearly observe the 3@SSN coating and original substrate. The superhydrophobic COMs 3@SSN with water contact angles (CAs) was up to 150.1° (Fig. 3c), exhibiting high oil–water separation efficiency of >99.5%, the highest among the advanced membranes.

Liu et al. synthesized free-standing COMs (SCOFs) at the expense of the bridge layer without destroying the COF framework [50]. Specifically, a self-assembled monolayer (SAM) was first prepared (Fig. 3d). Then, the SAM-modified silicon wafer was immersed in 1,3,5-triformylphloroglucinol (TFP) and 2,5-diaminobenzenesulfonic acid (Pa-SO_3_H) solution. The -NH_2_ of SAM caused the surface-initiated aldimine condensation polymerization of Tp and Pa-SO_3_H, leading to the formation of a sulfonic-acid-based COF layer on the substrate. Then, a bright yellow polymer was formed on the SAM after polymerization. Therefore, a free-standing SCOF was acquired after eliminating the sacrificing layer (Fig. 3e). Similarly, Zhang et al. developed a simple *in situ* molecularly soldered method to prepare thin COMs with outstanding sieving abilities through dopamine self-polymerization [51]. This work provided important guidance for the preparation of independent COMs.

Most COMs are assembled by simultaneous polymerization and crystallization in the liquid phase, which often results in a less ordered structure. Jiang et al. proposed a two-step method using a phase conversion strategy that decoupled the polymerization and crystallization processes to prepare highly crystalline COMs [52]. As shown in Fig. 3f, a mixed solution (aldehyde and amine) was first poured onto an indium tin oxide (ITO) substrate in order to generate an original membrane after evaporating the solvent. Then the reversibility of imine linkages at 60 ​°C was investigated. The obtained membrane was next transformed into COMs through the linkage rearrangement in vapor phase containing solvents. Free-standing COMs were obtained by etching the ITO (Fig. 3g).

### 2.3 Interfacial polymerization

**Fig. 4:**
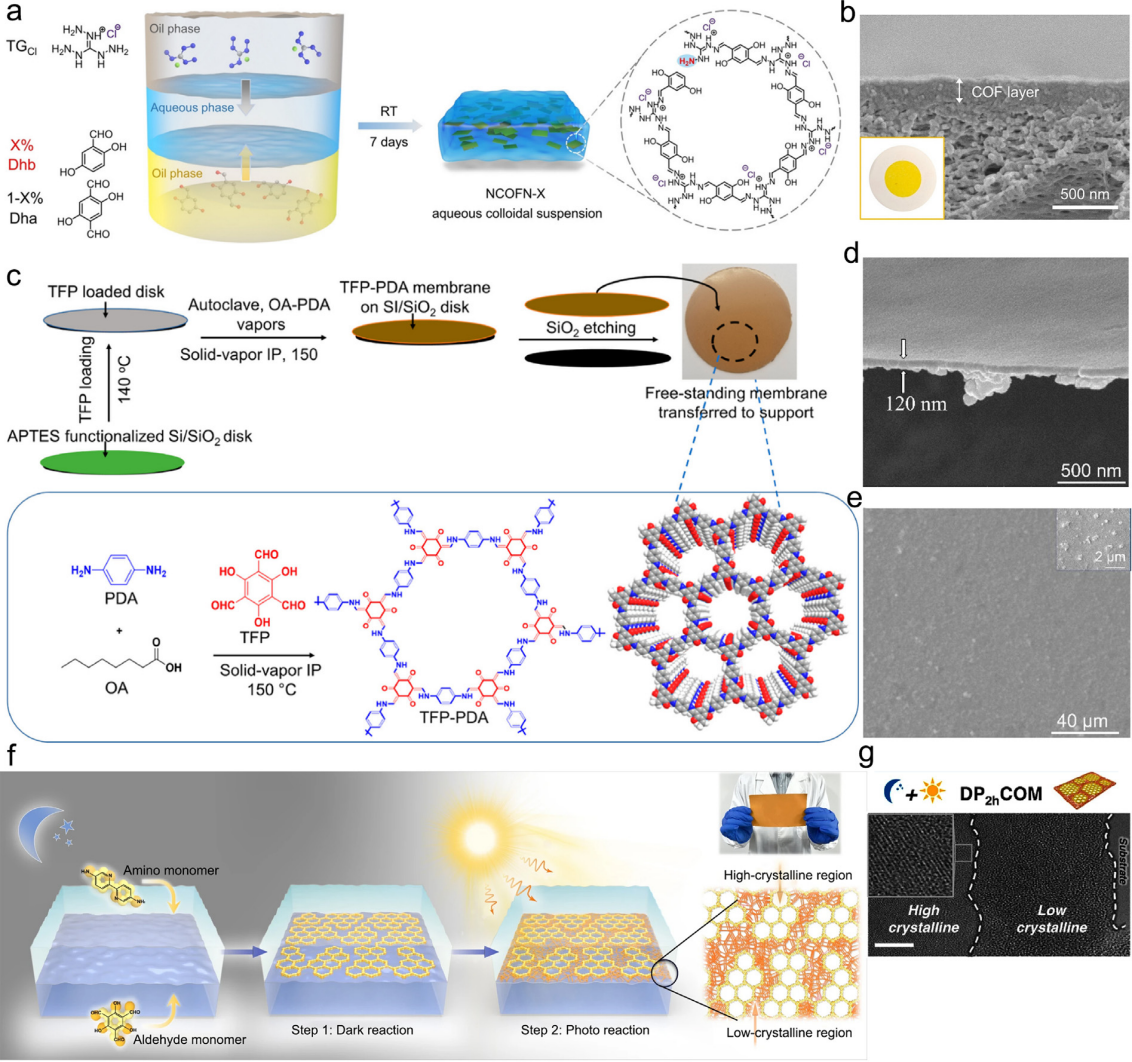
COMs preparation by interfacial polymerization strategy. (**a**) Schematic illustration of the synthesis process of NCOFN nanosheets; (**b**) cross-sectional SEM image and photographic image (inset) of COMs with layer thickness of 200 ​nm [55]; (**c**) the preparation process to obtain free-standing membrane by solid-vapor IP; SEM images of TFP-PDA membrane (**d**) cross-section, and (**e**) surface [58]; (**f**) preparation of heterocrystalline COMs by subsequent dark reaction and photo reaction using IP; (**g**) TEM image of COMs [61]. Panels reprinted with permission from: a–b, ref. [55], John Wiley and Sons; c–e, ref. [58], American Chemical Society; f–g, ref. [61], Springer Nature. IP, interfacial polymerization; NCOFN, amino-functionalization COF nanosheets; PDA, polydopamine; TEM, transmission electron microscopy; TFP, 1,3,5-triformylphloroglucinol.

IP, ​with exceptional designability, scalability, and applicability, is one of the most common methods for COMs preparation. The concept of synthesis of thin COMs by IP is to use the interface as a reactor, and the precursors dissolved in different phases diffuse to the interface and polymerize and crystallize to form thin films. As a pioneering fabrication, IP was first reported to synthesize continuous COMs in 2017 [53]. In this research, the organic linkers were dispersed in water and dichloromethane, respectively. The final thin membrane was produced at the interface of the oil and aqueous phase. With the continuous exploration and progress of COMs synthesis methods, an oil–water–oil tri-phase technique based on phase engineering was also reported. The intermediate water phase is a reaction region, and the two oil phases are storage zones to store and supply monomer molecules for the water phase. The big water space and relatively low precursor concentration result in the anisotropic gradual growth of COMs, which have the characteristics of thin thickness, large lateral size, and ultrahigh crystallinity [54]. As shown in Fig. 4a, the monomers of amine and aldehyde spontaneously diffused from the oil phase to the intermediate aqueous phase, the imine polymerization-crystallization occurred under the catalysis of acetic acid, and the aqueous phase offered a beneficial reaction region for the anisotropic growth of amino-functionalization COF nanosheets (NCOFN) [55]. NCOFNX (X ​= ​0, 10, 30, and 50) in the aqueous phase were obtained and further purified with deionized water. Plentiful -NH_2_ was produced on the COF nanosheets due to the linker defects in the assembly process of amine and aldehyde monomers. Subsequently, the obtained COF nanosheets were assembled to form COMs by filtration on polyacrylonitrile (PAN) substrates. The as-prepared COMs thickness of about 200 ​nm was observed from the SEM image (Fig. 4b). Hao et al. successfully synthesized thin COMs at the oil/water/hydrogel interface by loading both monomers into oil and hydrogel separately [56]. Moreover, this strategy exhibited high application potential for membrane preparation that could extend to the preparation of crystalline zeolitic imidazolate framework-8 (ZIF-8) membrane.

Volatile organic solvents were always used in liquid-liquid IP; however, solvent evaporation may destruct the liquid-liquid interface, affecting the crystalline structure of COMs. The domain size of the organic solvent-water interface is hardly regulated, while diffusion control of monomers can only achieve good crystallinity in the aqueous phase. Based on the above problems, Wang et al. synthesized a series of imine-linked COMs with different morphologies and thicknesses morphologies at the adjustable ionic liquid (IL)–H_2_O interface [57]. Due to the hydrogen-bonding of the catalysts between amine and the high viscosity of the ILs, the diffusion of the monomers was controlled in both water and ILs. This led to the unusually high crystallinity of obtained COMs with a surface area up to 4.3 times that fabricated at a dichloromethane–H_2_O interface. Long processing time is another disadvantage in the preparation of COMs by liquid-liquid interface polymerization. If this time lapse is shortened *via* improving the reaction temperature, it can cause damage to the fragile liquid-liquid interface, resulting in defects for the COMs. An engineered solid-vapor IP was first reported to fabricate highly crystalline COMs with ∼120 ​nm thickness within 9 ​h, much shorter than the time reported in the literature [58]. Fig. 4c shows the preparation process of COMs by the solid–vapor IP method. The Si/SiO_2_ disk was first modified with (3-aminopropyl)triethoxysilane (APTES). The TEP was dissolved in octanoic acid (OA) solution and then uniformly loaded on APTES- modified Si/SiO_2_ disk at 140 ​°C until the OA was all evaporated, forming a homogeneous TFP ​layer. Next, ​the polydopamine (PDA) solution was placed in the OA and the disk was loaded with TFP in an autoclave without direct contact. The ​ autoclave was heated to 150 ​°C until the PDA starts to evaporate with OA and then reacted with TFP at the solid-gas interface. The obtained membrane was then etched with 1% hydrofluoric acid to remove SiO_2_ layers to obtain independent COMs (TEP-PDA). Fig. 4d shows the cross-section SEM images of COMs with a thickness of 120 ​nm. The surface of COMs was uniform and smooth without large particles (Fig. 4e). For liquid-liquid IP, monomers was selected according to their solubility in their respective solvents, and for solid-gas IP, monomers was selected based on their melting point. For solid-gas IP, in order to avoid interface damage, the melting point of the solid phase monomer should be higher than the reaction temperature, and monomers with lower melting points should be used as the gas phase. Solid–vapor IP presents two obvious advantages compared with liquid-liquid IP: (I) Without disturbing the interface, it is feasible to accelerate the reaction rate by promoting the reaction temperature. (II) Since the monomers are in the static solid phase, it is easy to limit the reaction on the interface.

To date, low-crystalline COMs with much less ordered structures are much easier to fabricate, but it is quite difficult to obtain defect-free COMs with sufficiently high crystallinity [59,60]. In response to this problem, Yuan et al. successfully fabricated heterocrystalline COMs with continuous high- and low-crystalline regions through dark reaction and photo reaction [61]. The schematic preparation of heterocrystalline COM is shown in Figs. 4f; 2,2′-bipyridine-5,5′-diamine (Bpy) and Tp were selected as organic monomer to prepare COM in the dark reaction followed by photo reaction. High-crystalline COM was formed in the first dark reaction, while in the following photo reaction, a low-crystalline region in the defect of the COM was generated that flexibly connected the high-crystalline region and congested the defect (Fig. 4g). In the dark conditions, the reversible enol-imine linkage led to the high-crystalline COM formation and the subsequent introduction of photo irradiation allowing the low-crystalline region formation in the intercrystalline defects region. By adjusting the photo reaction time, the low-crystalline region and the high-crystalline region can be tightly and flexibly connected, which helps obtain defect-free COM. Moreover, it was experimentally confirmed that the low crystalline region grew in the intercrystalline defects rather than along the film thickness direction.

### 2.4 Electrochemical synthesis

The electrochemical technique, featuring with high efficiency, simple operation, environmental friendliness, and precise controllability, is a useful strategy for directly preparing thin-film material on electrodes [62]. Besides, the controlled energy input from the electrodes makes it possible to control chemical reactions in a pre-designed way. To date, menthods, such as electro-deposition, electrochemical IP, electrocleavage synthesis strategy, and potential difference-modulated biphasic strategy have been reported for preparing COMs [63,64].

**Fig. 5:**
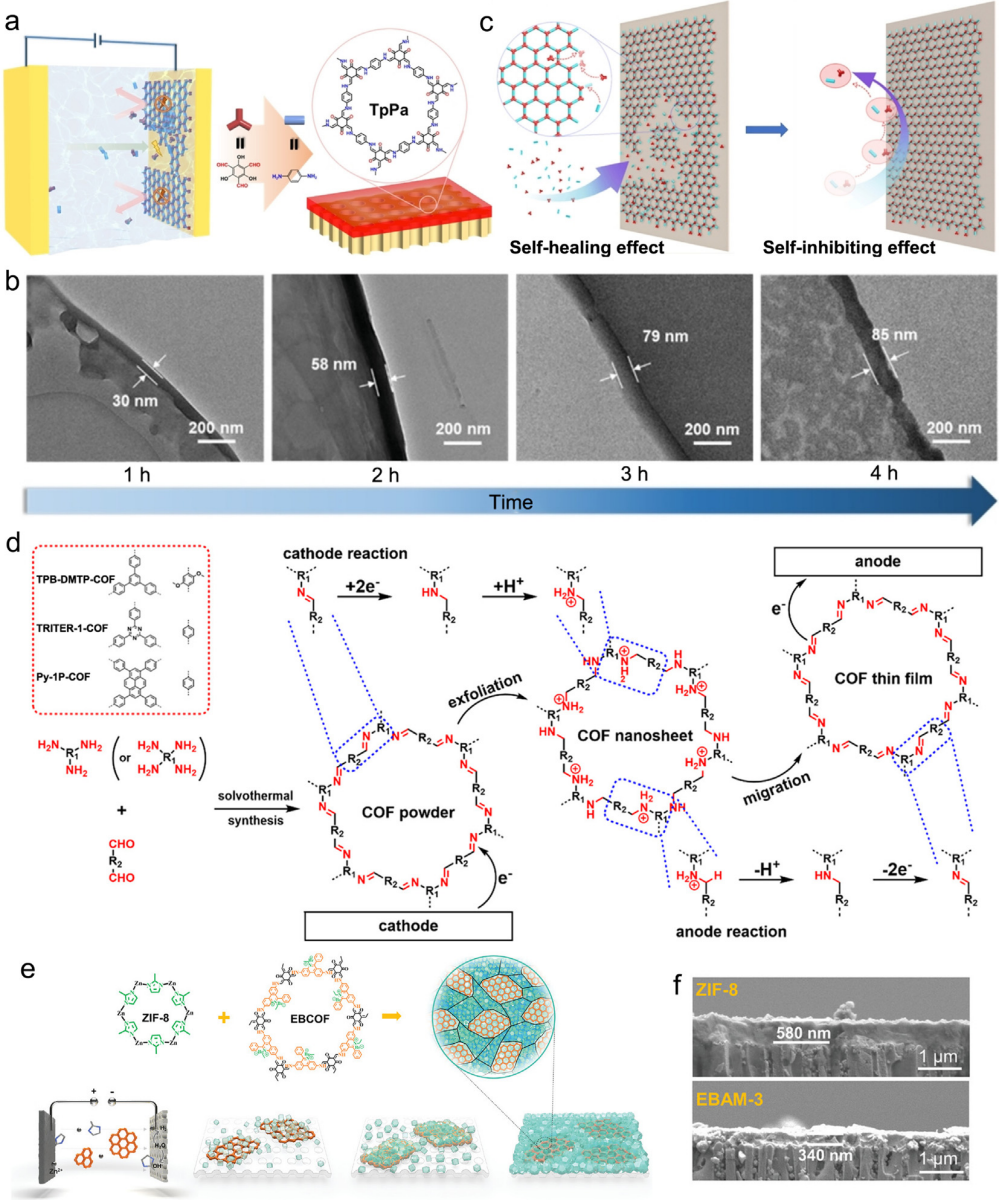
COMs preparation by electrochemical synthesis. (**a**) The synthesis diagram of ultrathin COMs by electrochemical IP; (**b**) cross-sectional TEM images of TpPa membranes with different electrochemical time; (**c**) the self-healing effect and the self-inhibiting effect [63]; (**d**) total electrode reactions in the electrocleavage synthesis process [37]; (**e**) schematic for the fabrication process of AMs; (**f**) cross-sectional SEM images of ZIF-8 membrane (top) and EBAM-3 (bottom) [71]. Panels reprinted with permission from: a–c, ref. [63], John Wiley and Sons; d, ref. [37], American Chemical Society; e–f, ref. [71], John Wiley and Sons. AM, “alloy” membrane; ZIF-8, zeolitic imidazolate framework-8.

Electrochemical technique not only offers more options for COMs preparation, but exhibits unique advantages compared with other exploited methods. IP is the most commonly used method to prepare COMs, despite some problems such as much longer required time, tedious solvent optimization steps, and poor stability [42,[65], [66], [67], [68]]. More importantly, to acquire ultrathin COMs by IP is still a major challenge. For this problem, Wang et al. fabricated ultrathin COMs with a thickness of 85 ​nm through electrochemical IP (Fig. 5a) [63]. Organic monomer Tp and p-phenylenediamine (Pa) dispersed in methanol and moved to the cathode under the control of electric field, resulting in the formation of ultrathin COMs. Fig. 5b exhibited the variation of COMs thickness with reaction time; the thickness of COMs was maintained at 85 ​nm and almost unchanged when the reaction time exceeded 4 ​h. The mechanism of ultrathin COMs preparation by electrochemical IP was also described in detail. The electrochemical process showed self-healing and self-inhibiting toward the preparation of thin and continuous COMs [69,70]. The higher voltages can divide polymerization reactions into reaction-controlled region and migration-controlled region. At the reaction-controlled region, electrochemical reaction plays the role of monomer migration and intermediate deprotonation, and the thickness of the COMs increases rapidly as the defect heals. In migration-controlled region, the generated COMs serve as a barrier to prevent migration of electrons (Fig. 5c). Therefore, electrochemistry only promotes the migration of monomers ​and has little effect on the increase of membrane thickness.

Apart from electrochemical IP, an electrocleavage synthesis strategy was also developed and applied to prepare imine-linked COMs on electrodes from electrolyte solutions [37]. This strategy used electrochemical reduction and protonation to strip the COF powder into nanosheets at the cathode, which then migrated to the anode and reproduced the COF structure by oxidation. As shown in Fig. 5d, cathodic reduction transferred imine bonds to protonated amine bonds and introduced a large positive charge to the COF framework, striping COFs to abundant nanosheets with high dispersion. Then, the formed COF nanosheets moved to the counter electrodes under the electric fields and underwent oxidation to recover the imine bonds from the protonated amine bonds and reproduce thin crystalline COMs on the electrodes. This synthesis is suitable for most imine-linked COFs, and features simple and fast operation, eco-friendliness, and mild preparation conditions, providing some guidance for preparing high-quality COMs.

Recently, a potential difference-modulated biphasic method was reported to synthesize continuous and independent COMs [64]. This synthetic process was performed at the interface of polarized water/1,2-dichloroethane (water/DCE), where HCl acted as a catalyst in water solution and monomers molecule (both amine and aldehyde) were added to DCE. By cyclic voltammetry, the external polarization at the water/DCE interface continuously pumped H^+^ from water into DCE, promoting the Schiff base reaction of monomers and the formation of COMs. Three types of centimeter-scale, independent COMs with uniform pore size, functional surface and defect-free structure were prepared *via* this strategy. Besides single COMs, “alloy” membranes (AMs) consisting of COMs and MOFs were successfully fabricated through reductive electrosynthesis and electrophoretic deposition [71]. First, COF nanosheets containing ethidium bromide units were preliminarily synthesized through interfacial crystallization [69]; the formed COF nanosheets were then added to the ZIF-8 synthesis process for an electrically driven co-deposition. The ZIF-8 nuclei were formed *via* the coordination of Zn^2+^ ions with reduced de-proton ligands in the cathode or solution. Positively charged COF nanosheets, and ZIF-8 nuclei in solution are electrophoresed onto the cathode to form AMs with a mixed matrix structure (Figs. 5e and f).

Electrochemical technology provides more options for the preparation of COMs; ​more importantly, it features enormous advantages, such as simple operation, avoiding tedious optimizations of solvent, and a relatively fast preparation process. Because of the precise control of the synthesis process, the COMs prepared by electrochemical synthesis technology show enhanced stability, defect-free structure, and ultrathin thickness.

### 2.5 Other methods

In addition to the above methods used to prepare COMs, other methods such as vacuum-assisted layer-by-layer method, filtration method, and compression method have also been reported to fabricate COMs [38,72]. Wang et al. prepared three different acid-based modified COMs (i.e., phosphoric acid, sulfonic acid, and carboxylic acid) in the channels by the vacuum-assisted layer-by-layer method [72]. The diamine and aldehyde monomers first diffused in the opposite direction, meeting in intermediate water, and triggering the reactive assembly of COF nanosheets. Subsequently, the obtained COF nanosheets formed membranes through filtration. SEM images confirmed that the as-prepared COMs had uniformly dark-red morphologies, without obvious pinholes or cracks. Lu et al. prepared COMs with high stability, permeability, mechanical property, and separation performance through the vacuum-assisted layer-by-layer method [38]. The as-prepared COMs exhibited a high rejection rate (>99%) to methylene blue. Compression, a simple method, was reported to prepare free-standing homogeneous, large-scale, and porous imine-based COMs with excellent mechanical properties by compression of imine-based COF-aerogels [73]. This method could produce COMs with diameters ranging from 1 to 5 ​cm and thicknesses of 50–60 ​μm, which were thinner than previously reported COMs [74].

Disorder-to-order transformation is emerging as a powerful and attractive strategy for fabrication membranes [[75], [76], [77], [78], [79], [80], [81]]. Jiang et al. first used the disorder-to-order transformation method to prepare imine-linked COMs with high crystallinity from amorphous polyimine membranes through monomer exchange based on dynamic covalent chemistry (DCC) [82]. Amorphous polymeric membrane 1,3,5-tris-(4-amidophenyl)triazine and 1,4-phthalaldehyde (TAPT–PA) was facilely prepared by heating the solution that contained TAPT and PA. Then, 2,5-dihydroxyterephthaldeyde (DHTA) was chosen as the replacing monomer because TAPT–DHTA was more stable than TAPT–PA. Finally, the conversion of amorphous-to-crystalline membrane was realized after 72 ​h. After solvothermal treatment, the yellow TAPT–PA membranes turned red. Multiple testing instruments, such as Fourier transform infrared (FT-IR), X-ray diffraction (XRD), ^13^C nuclear magnetic resonance (NMR) spectra, were further executed to verify the formation of COMs TAPT–DHTA that featured abundant porosity, high crystallinity, and complete structure. The mechanism of the disordered-to-ordered membrane transformation was proposed, DHTA monomers are nucleophilically attacked by the imine bond of the amorphous TPT–PA membrane catalyzed by acetic acid [83]. Then, PA monomers were exchanged by DHTA monomers *in situ*, condensing between TPT and DHTA to form new imine bonds, and amorphous TAPT–PA started to translate crystalline TAPT–DHTA driven by the minimization of Gibbs free energy, achieving the co-existence both crystalline TAPT–DHTA and amorphous TAPT–PA with time. As the reaction progressed, all the amorphous TAPT–PA converted into a highly crystalline TAPT–DHTA membrane. Following the principle of DCC, Giri et al. achieved direct conversion of discrete organic imine cages-to-COMs on the liquid-liquid interface at ∼25 ​°C [84]. Continuous structural reorganization, such as the unfolding of the imine cage, resulted in the COMs growth. The whole process included (i) the unfolding of the imine cage resulting in the formation of imine intermediates (ImIs), (ii) a disordered network formed as a dynamic intermediate at the interface, and finally, (iii) the DCC-directed “error correction” leading to the formed crystalline COMs.

This section detailly summarizes the latest preparation methods for COMs, including layer-by-layer stacking, *in situ* growth, IP, electrochemical synthesis, filtration method, compression method, and so on. For water treatment, COMs prepared by the above method possess excellent structure integrity, high crystallinity, hierarchical pores with high surface areas, and robust stability, ​and are touted as ideal materials for seawater desalination, toxic ions and organic contaminants removal, and oily water separation. *In situ* growth is a widely used method for preparing COMs supported on substrates. However, due to the poor controllability of heterogeneous nucleation on the substrate surface, the manufacture of large-area COMs remains difficult. Therefore, the stability of COMs prepared by *in situ* growth method needs to be further improved. IP is the representative of synthesis of free-standing and continuous COMs. This traditional water-organic IP process has been widely used to prepare COFs composite membranes on polymeric substrates. The sieving action of COMs is very important for seawater desalination and organic contaminant separation. Therefore, COMs with target aperture can be designed for different sizes of organic dyes to achieve their efficient interception. To improve the performance of selective capture metal ions, a common method is to modify COMs with chelation groups. Alternatively, electrochemical synthesis with high-efficiency, simple operation, environmental friendliness, and precise-controllability is a useful strategy for preparing thin-film material directly on electrodes. Besides, the controlled synthesis process avoids tedious steps and the use of large amounts of solvents. From the perspective of cost and environmental protection, the electrochemical preparation of COMs is a promising method.

Compared with other preparation methods, *in situ* growth, layer-by-layer stacking, and IP can produce continuous COMs, achieving the full function of COFs in separations. In addition, ultrathin COMs prepared by layer-by-layer stacking and IP demonstrate superhigh permeability, and enabling these two methods is very promising for the future application of COMs. For specific target applications, the preparation method of COMs can be combined with the following strategies to improve their performance in the target application. Hydrogen bonds can be introduced into the COF backbone or compounded with other materials to improve stability. The separation and removal of organic dyes depend on the pore size of COMs, so a high retention rate of target dye molecules can be achieved by adjusting the aperture size of COF during the synthesis of COMs. To achieve efficient separation efficiency of metal ions, COFs need to modify with specific groups, and then efficient removal of metal ions by COMs through coordination, electrostatic, or chelation can be achieved.

## 3 Characterization techniques for COMs

The properties of the COMs, such as the ordered structure, regular pore size, high pore density, large specific surface area, and tailored functionality, require a variety of characterization test results to verify. Common characterization testing techniques are necessary for COMs, such as powder XRD, FT-IR, X-ray photoelectron spectroscopy, Brunauer-Emmett-Teller, ^13^C cross-polarization magic angle spinning (CP-MAS) NMR spectrum (^13^C CP/MAS NMR), SEM, and high-resolution transmission electron microscopy (HRTEM)[85]. Besides the above mentioned, atomic force microscope (AFM), water CA, small angle X-ray scattering/wide angle X-ray scattering (SAXS/WAXS), and grazing incidence small angle X-ray scattering/grazing incidence wide angle X-ray scattering (GISAXS/GIWAXS) ​are always applied for investigating the peculiarity of COMs.

**Fig. 6:**
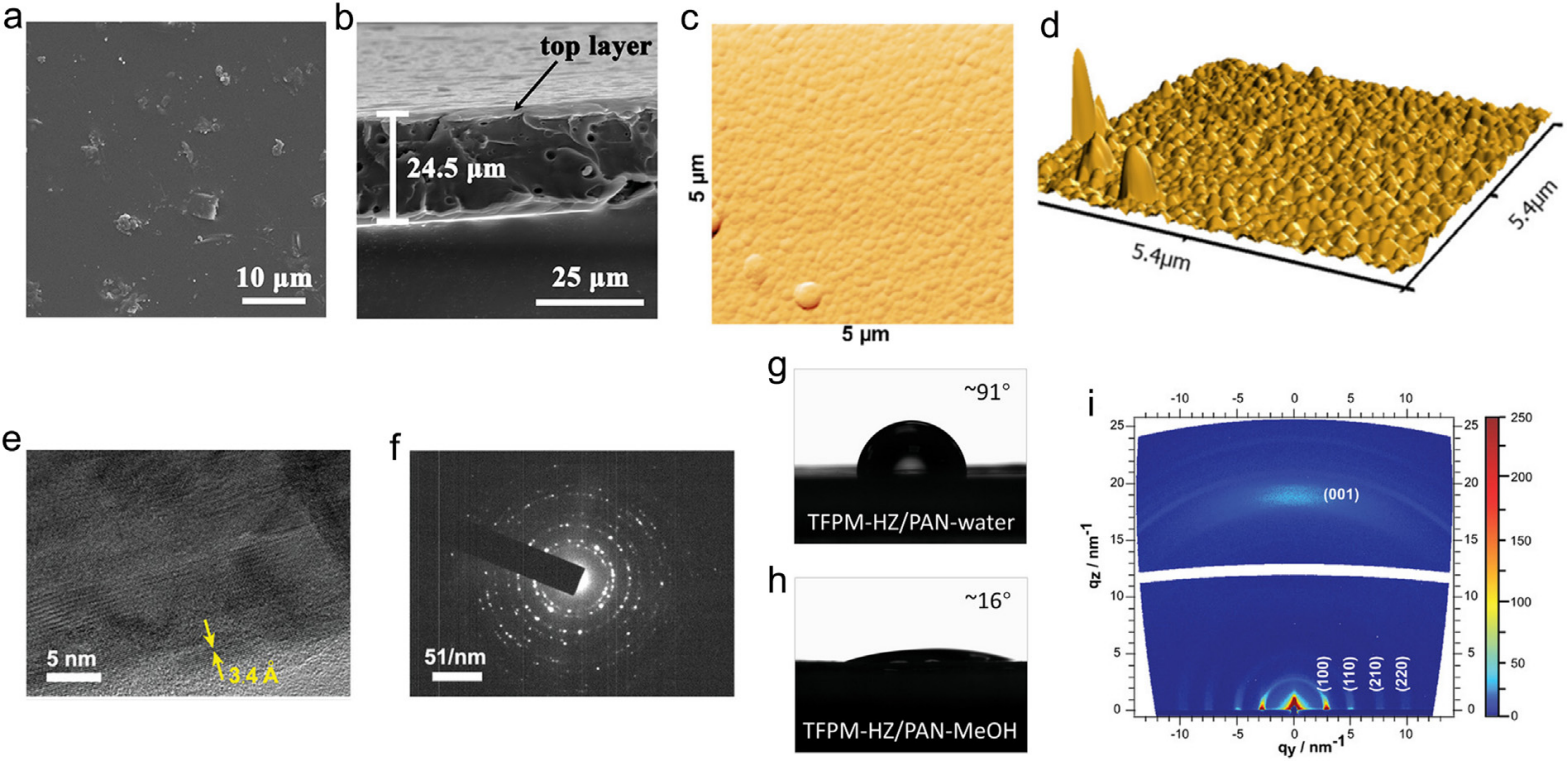
Various characterization techniques for COMs. SEM images of (**a**) top side surface and (**b**) cross-section of CD-COF-1 [34]; (**c**) AFM image of 5 ​× ​5 ​μm area and (**d**) 3D depiction of the film topography [48]; (**e**) HRTEM of TpBDMe_2_ and (**f**) SAED of TpBDMe_2_ confirming the high crystallinity of the synthesized COF [86]; (**g**, **h**) solvent contact angles of the support and membrane [87]; (**i**) GIWAXS 2D patterns of BTT TTA thin film grown on ITO [44]. Panels reprinted with permission from: a–b, ref. [34], John Wiley and Sons; c–d, ref. [48], American Chemical Society; e–f, ref. [86], John Wiley and Sons; g–h, ref. [87], John Wiley and Sons; i, ref. [44], John Wiley and Sons. AFM, atomic force microscope; BTT, benzotrithiophene; CD, cyclodextrin; GIWAXS 2D, grazing incidence wide angle X-ray scattering; HRTEM, high resolution transmission electron microscope; SAED, selected-area electron diffraction; TTA, triazine-based amines (1,3,5-triazine-2,4,6-triyl)trianiline.

SEM is a characterization mean used to observe the microscopic morphology of nanomaterials, which feature a large field of view and good stereo imaging effect. SEM characterization technology can be used to observe the surface morphology of the obtained COMs and the cross-sectional thickness of the prepared COMs, and further evaluate the quality of the prepared COMs. For COMs, cross-section SEM images and top-view SEM images are frequently used to observe the thickness and analysis of the surface smoothness of COMs. Figs. 6a and b are the SEM images of COMs CD-COF-1 MMM. Then SEM images of top side showed that particles are evenly distributed on the surface of CD-COF-1 MMM (Fig. 6a). The thickness of the membrane was ∼24.5 ​μm, as observed from the cross-section image (Fig. 6b) [34].

AFM is one of the most powerful tools for the surface analysis of materials, which can be used to observe the surface of COMs, size measurement, surface roughness measurement, film formation condition evaluation, and so on. For example, Medina et al. used AFM to assess the surface roughness and morphology of COMs. The AFM scan displays a surface comprising an intergrown domains, forming a continuous, almost perfect thin membrane (Figs. 6c and d). The average roughness extracted from the film topography is ∼6 ​nm, which is a relatively smooth surface [48].

Compared with SEM and TEM, HRTEM possesses higher resolution and can clearly observe the lattice fringes and regular pore size of COFs. By measuring the fringe spacing, the crystal plane is determined by comparing it with the standard crystal plane spacing d, thus determining the crystal orientation or the growth direction of the material. Sheng et al. observed that the distance between adjacent lattice fringes was 3.4 ​Å of COMs TpBDMe_2_, which was consistent with the theoretical interlamellar spacing (Fig. 6e), indicating that the as-prepared COMs feature highly-ordered arrangement and high crystallinity. Selected-area electron diffraction ​pattern (Fig. 6f) showed diffraction points, further corroborating the high crystallinity of the synthesized COMs [86].

CA ​is one of the powerful tools used to explore the hydrophilicity and hydrophobicity of materials. A CA < 90° indicates that the material is hydrophilic, while >90° is hydrophobic. By exploring the CA of COMs, it can provide valuable references for developing its potential application. Shi et al. investigated the wettability of COMs TFPM-HZ/PAN to water and methanol. Interestingly, although TFPM-HZ/PAN has an angstrom-grade channel, it has an extremely high affinity for methanol and can quickly penetrate methanol within 2 ​s (Figs. 6g and h) [87]. The results displayed that the TFPM-HZ/PAN backbone showed hydrophobicity and excellent wettability to methanol. Thus, the sub-nanometer pore and beneficial organic solvent penetration make TFPM-HZ/PAN a potential application prospect in high-performance organic solvent nanofiltration.

GISAXS (grazing incident small-angle X-ray scattering) and GIWAXS (grazing incident wide-angle X-ray scattering) are two types of small angle X-ray scattering. Through analysis of the obtained GISAXS and GIWAXS image, the geometric dimension of the sample in the nanoscale range (1–100 ​nm) can be obtained, including the shape, radius of rotation, average particle size, average wall thickness, molecular weight, porosity, and other structural information. For COMs, GISAXS and GIWAXS are powerful instruments to examine the crystallinity of the membrane and verify the optimal orientation of the COF crystallites [88]. Frey et al. conducted a detailed analysis for COMs BTT TTA using GIWAXS [44]. As shown in Fig. 6i, the distinct reflections at *q*_*y*_ ​= ​2.78, 4.89, 7.56, 9.82 ​nm^−1^, and *q*_*z*_ ​= ​19.13 ​nm^−1^ are clearly observed, which corresponded to *hkl* (100), (110), (210), (220), (001), and the *q*-values acquired by data-reduction to 1D-plots are consistent with the XRD patterns of those which obtained bulk powder. The GIWAXS data of thin COMs proved the optimal orientation with the [001]-axis (c-axis) to be oriented orthogonal to the surface.

The characterization methods can not only explore the properties of COMs, which is also conducive to the design of COMs materials for target applications, and can also explore the changes and microscopic mechanisms before and after the reaction of materials. With the aim of deepening understanding of COMs, more advanced characterization techniques have been applied to further study the microstructure and peculiarity, developing its potential advantages in target applications.

## 4 Application of COMs in water purification

COFs feature low density, adjustable element composition, super specific surface area, adjustable pore size and microstructure, and facile functionalization, arousing extensive research interests in multi-field. These characteristics make COFs a new-generation nanomaterial in water purification including heavy metal removal [89], dye separation, radionuclides purification, pollutant detection, seawater desalination, and ion fractionation, etc. (Table 1) [90]. For water treatment, the specialties of stability, aperture size, and surface functionalization of COFs and COMs are very important. To improve the performance of COFs materials in water treatment applications, stability is an obvious prerequisite. Strategies to enhance the stability include the introduction of intramolecular hydrogen bonds, organic linkage conversion, and the construction of hybrids with other water stable materials. The pore size of COFs plays a significant role in the water treatment application, especially for COMs separation technology. The pore size of COFs is mainly distributed in the range of 0.5 ​nm–4.7 ​nm. Depending on the aperture effect and the target application, pollutants with different sizes can be trapped and separated. Two methods can be used to fabricate COFs with specific pore sizes. One is the direct preparation of COFs with specific apertures through carefully selecting organic linkers with specific lengths. The other method is to modify side groups to the COF channel structure to regulate pore size. Surface functionalization can endow COFs with new or modified properties such as hydrophilicity and charge distribution. In addition, modification of the chelation groups to COFs can significantly enhance the affinity for the target metal ion. The complex water system requires that COFs materials must consider the water stability, surface properties, hydrophilicity, and charge distribution.

**Table 1:** The application of COMs in water treatment.

Materials	Application	Performance	Ref.
CF@TpTph	Removal of Cd^2+^	84.1 mg/g	[91]
TFN HF NF membrane	Heavy metal separation	95.4% for Cr_2_(SO_4_)_3_ 94.3% for CuSO_4_ 91.7% for ZnSO_4_ 90.9% for MnSO_4_	[89]
	Dye solution separation	98.7% for rose bengal 97.2% for coomassie brilliant blue; 93.5% for indigo carmine 90.8% for safranine T	
TpPa-SO_3_Na	Dye separation	96% for cationic dyes	[93]
TpTAPA/HPAN	Dye separation	>92.0%	[92]
TAPB-PDA COF pellet	Dye separation	100% for rhodamine B	[94]
TPB-DMTP-COF	Iodine adsorption	6.37 g/g	[37]
[NH_4_]^+^[COFSO_3_^−^]/SPES MMMs	Uranium extraction	99.4 mg/g	[95]
TaPa-SO_3_H nanosheets	Seawater desalination	rejection of NaCl 99.91%, water flux of 267 kg/(m^2^·h)	[41]
TpHz	Desalination	Na_2_SO_4_ rejection of 58.3% water permeance of 40.5 L/(m^2^·h·MPa)	[66]
COF@SSN	Oil–water separation	99.5%, permeation flux of 2.84 ​× ​10^5^ L/(m^2^·h)	[49]

CF, carbon fiber; DMTP, dimethoxyterephthaldehyde; HPAN, hydrolyzed polyacrylonitrile; MMMs, mixed matrix membranes; SPES, sulfonated-polyethersulfone; TAPA, tris(4-aminophenyl)amine; TAPB, 1,3,5-tris(4-aminophenyl)benzene; TFN HF NF, thin film nanocomposite hollow fiber nanofiltration; Tp, 1,3,5-triformylphloroglucinol; TPB, triphenylbenzene.

### 4.1 Removal of metal ions

Jin et al. reported a porphyrin-based COMs CF@TpTph *via in-situ* growth, and the interlaminar porphyrin molecules are stacked vertically [91]. This vertical stacking configuration in space expanded the central space of the porphyrin ring, promoting the coordination of Cd(II) with porphyrin ring center and endowing the COF with the dual functions for ratiometric detection of Cd(II) and elimination of Cd(II) as adsorbent. The maximum adsorption capacity of CF@TpTph for Cd(II) was 75 mg/g, and the adsorption equilibrium was reached within 40 ​min. In addition, the adsorption behavior of Cd(II) by CF@TpTph membrane was studied in the coexistence of various competing ions. The presence of competing ions had very little effect on the adsorption of Cd(II) by the CF@TpTph membrane, and >90% removal efficiency was maintained due to the strong Cd(II) coordination ability and fast Cd(II) adsorption kinetics of CF@TpTph membrane. The density functional theory results showed that Cd(II) formed a stable structure with the four N complexes in the center of the porphyrin ring, which promoted the removal efficiency of Cd by CF@TpTph. This study revealed that the design of chelating groups in COFs with precise coordination with metal ions can greatly improve the removal performance.

### 4.2 Removal of organic dyes

**Fig. 7:**
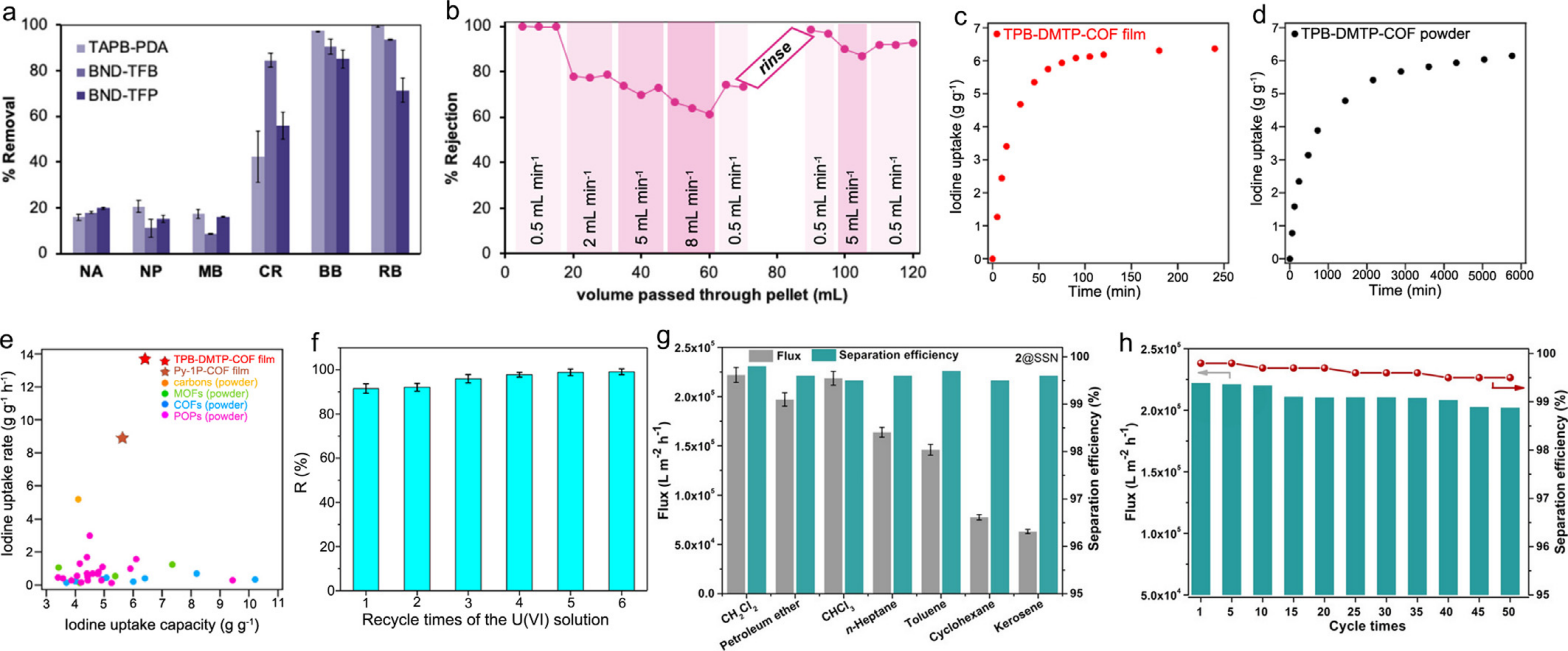
The application of COMs in water treatment. (**a**) Removal of various organic dyes (50 mg/L) from Milli-Q water by three COFs (333 mg/L); (**b**) plot of rejection of RB as a function of total passed volume with variable flow rate [94]; (**c**) iodine uptake of the TPB-DMTP-COF film as a function of exposure time at 350 ​K and ambient pressure; (**d**) iodine uptake of the TPB-DMTP-COF powder as a function of exposure time at 350 ​K and ambient pressure; (**e**) iodine uptake capacities and rates of different adsorbents [37]; (**f**) effect of recycle times of the uranium solution on uranium removal efficiency [95]; (**g**) separation efficiency and oil flux of 2@SSN coatings for different oil/water mixture systems; (**h**) cycle tests of oil flux and separation efficiency of 2@SSN coatings for the separation of CH_2_Cl_2_/water [49]. Panels reprinted with permission from: a–b, ref. [94], American Chemical Society; c–e, ref. [37], American Chemical Society; f, ref. [95], Elsevier; g–h, ref. [49], John Wiley and Sons. RB, rhodamine B.

Polycrystalline COMs have been applied to separate dyes and other pollutants based on size-selective transport by ordered pores. Zhao et al. fabricated triphenylamine-based COMs TpTAPA/HPAN by an oligomer-triggered interfacial polymerization (OT-IP) ​and observed the OT-IP process through an optical *in situ* device [92]. The as-prepared TpTAPA/HPAN showed a water permeance of 68.1 L/(m^2^h·bar), and the rejections of dye eriochrome black T, congo red ​and drug diammonium glycyrrhizinate, ammonium glycyrrhizinate ​above 92.0%. Due to the electrostatic attraction, an effective strategy is to construct ionic COMs for efficient dye removal. Yang et al. prepared anionic COMs TpPa-SO_3_Na through dual-activation IP and applied it to separate cationic dyes [93]. Because of the electrostatic interaction between positively charged dyes and anionic COMs channels, the removal efficiency of multiple cationic dyes was >96% by TpPa-SO_3_Na. COMs hows great promise in the separation of dyes; however, it is not clear whether the removal mechanism of dyes by COMs is adsorption behavior or membrane separation. Fenton et al. verified that the dye removal was consistent with an adsorption mechanism by COMs through experiments, not dependent separation mechanisms [94]. They first confirmed that the adsorption behavior of the three COFs to the dye pairs was similar (Fig. 7a), and the adsorption isotherms of different dyes by the three COFs were well-fitted. Next, “membrane” experiments are performed to investigate the removal effect off dye. Fig. 7b shows the change in rhodamine B (RB) removal rate with flow velocity. As flow increased, the retention rate decreased dramatically, from >99% rejection (0.5 mL/min) to 78% (2 mL/min), 70% (5 mL/min) ​and 65% (8 mL/min). This decreased rejection coincided with the adsorption mechanism, as the contact time between the dye and the sorbent decreased, so did the adsorption capacity. They then returned the flow rate to 0.5 mL/min, and the rejection immediately returned to 75%. The researchers washed the pellets with methanol and water to eliminate the adsorbed RB, and the removal performance was fully recovered (>95%). These results indicated that the dye removal behavior by COF was consistent with the adsorption mechanism. In addition to size effects, this study revealed the importance of designing sites with a high affinity for organic dyes in the removal of organic dyes by COMs.

### 4.3 Removal of radionuclides

COMs provided a superb platform for efficient removal of radionuclides. Wang et al. synthesized thin COMs through the electrocleavage method ​and used these COF films as adsorbent to remove iodine [37]. TPB-DMTP-COF films showed unusually fast adsorption, which increased sharply within 30 ​min, then saturated in 240 ​min, reaching an adsorption capacity of 6.37 g/g (Fig. 7c). This excellent adsorption performance contrasted with TB-DINTP-COF powder, which increased within 36 ​h and reached an adsorption capacity of 6.15 g/g in 96 ​h (Fig. 7d). The rate constant was up to 13.69 g/(g·h) for TPB-DMTP-COF, far beyond those of advanced iodine adsorbents (Fig. 7e). Wu et al. synthesized COF-based MMMs [NH_4_]^+^[COF-SO_3_^-^]/SPES and applied it to investigate the removal efficiency of uranium from acidic aqueous solutions [95]. Benefiting from strong adsorption sites (-SO_3_H) toward uranium(VI) and enhanced acid resistance, [NH_4_]^+^[COF-SO_3_^-^]/SPES demonstrated excellent adsorption capacity of 99.4 mg/g for uranium at pH 1. After five cycles, the removal efficiency of [NH_4_]^+^[COF-SO_3_^-^]/SPES to uranium remained >90%, demonstrating promising prospects in the appellation of recovery uranium from water (Fig. 7f). Similar to the mechanism of removing metal ions, the mechanism by which COMs remove radionuclides includes ion exchange, coordination interaction, chelation, and electrostatic interactions. In particular, for the separation and removal of radioactive gases such as iodine, designing the pore size and pore structure of COMs is the key to improving the removal kinetics.

### 4.4 Seawater desalination

Seawater desalination is considered a promising way for the continued supply of fresh water resources. It benefits from the intrinsic characteristics of COF and the properties that can be pre-designed functional groups, easily obtained COMs with good fouling resistance, high hydrophilic surface and high permeation flux. Wang et al. reported COMs with high water flux and strong scale resistance, achieving a permeation flux of 267 kg/(m^2^·h) at 50 ​°C for the 3.5% NaCl aqueous solution [41]. Moreover, the high desalination performance can be kept steady for 108 ​h, proving the durability and stability of COMs. XRD and FT-IR confirmed the crystal and chemical integrity of COMs after desalination. By pre-designing functional groups on the COFs skeleton to regulate their hydrophilicity and surface charge distribution, permeation flux and high rejection can be increased.

### 4.5 Oil–water separation

Oily water generated from industrial production has led to a huge threat to environmental protection, and developing an effective method for oil–water separation is urgent. COFs possess adjustable and designable nano-space, which provides different capabilities for the target application. Liu et al. broke through the inherent drawbacks of hydrophobic COFs as crystalline membranes in terms of wettability and processability ​and prepared robust COMs by introducing mixed hydrophobic substituents, achieving the purpose of ultra-high-speed oil–water separation [49]. Due to the large pore size and excellent lipophilicity of 2@SSN, all oil could pass through the coating quickly, with the oil flux of more than 0.60 ​× ​10^5^ L/(m^2^·h) for kerosene/water mixture (Fig. 7g). As the viscosity of the oil increased, the oil flux showed a downward trend. The as-prepared COMs 2@SSN exhibited average separation efficiency of 99.5% in 50 cycles of reuse, indicating that the COMs can be reused many times without any significant degradation of the separation efficiency (Fig. 7h).

This section introduces the application of COMs in water purification and analyzes its potential mechanism in detail. Compared with COF powder, COMs show broader potential in the purification of pollutants from water, seawater desalination and ion separation. Powder materials are not easy to collect in an aqueous solution, difficult to recycle and complicated to dispose. The preparation of powder COFs into the membrane avoids the above problems; moreover, the features of ordered pore size ​and functionalized pore structure of COMs may offer a direct transport channel through the pores for small molecules.

## 5 Conclusion and prospect

Recently, advanced porous nanomaterials such as MOFs, COPs ​and HOPs have been developed and have afforded a broad platform for application in water treatment. MOFs are a class of crystalline porous material with periodic network structure formed by self-assembly of inorganic metal center (metal ion or metal cluster) and bridged organic ligand, allowing versatile operation of the functional units and precise pore control, increasing the applicability of MOFs for water purification. Crystalline COFs are different from MOFs in that they are synthesized by purely organic monomers *via* more robust covalent bonds and possess the advantage of easy functionalization of building blocks and pore surfaces. COPs are porous nanomaterials with purely organic backbones but lack long-range order structure. Compared to MOFs COFs ​and COPs, it is more difficult to stabilize HOFs and establish their permanent porosities given the fact that hydrogen bonds are typically weaker than ionic, coordination ​and covalent bonds. Compared with other porous materials, COFs materials generally show stronger stability and easy functionalization. However, the high preparation cost limits the application of new porous materials including COFs in water treatment, which requires more simple and low-cost COF synthesis methods to be explored in future development.

As an emerging crystalline porous material, COFs consist of orderly arranged pore structures, which endow them large surface area, very low density ​and abundant porosity [96]. Moreover, the versatile organic connectomes used to synthesize COFs give them easily functional features. These features have made COFs promising candidate materials for membrane preparation. COMs show outstanding adsorption capacity and reusability for heavy metal ions and dyes, and exhibit excellent selectivity for target pollutants compared with other nanomaterials. COMs combined with other nanomaterials are an effective strategy to achieve synergistic effects for eliminating contaminants. Moreover, appropriate modification of functional groups in COMs channels can enhance the affinity for target pollutants. In addition, COMs with controllable channel size, high permeation flux, excellent sieving ability, and enhanced stability ​can show high separation efficiency for seawater desalination and oil–water separation.

Although remarkable achievements have been made in the preparation of COMs and their application in water treatment, some challenging issues remain to be addressed, which also provide great opportunities for researchers in this field. (I) To realize ultrafast and highly selective molecular sieving, COFs with ultra-microporous (<1 ​nm) should be constructed; (II) the poor stability performance and low permeability limit the practical applications of COMs during water treatment processes, and developing new synthetic strategies to synthesize high-performance COMs is highly urgent; (III) the mechanism of ion adsorption on COMs, the development of customized active sites for selective ion capture ​and the role played by COFs in the separation processes still need further investigation; (IV) due to the significant differences between industrial production and experimental environment, the long-term stability of COMs under practical separation conditions are still very limited; (V) further research can focus on 3D COFs because the pore size of 3D COFs is often small due to the existence of structural interpenetration.

However, the future development and potential application of COMs in academia and industry remain challenging. At present, the COMs preparation method has the disadvantages of a time-consuming process and complexity, and developing simple and green COMs synthesis techniques is highly required. In addition, research on COMs that can achieve large-scale preparation with independent stability has not been reported. For water treatment applications, COMs with long-term stability, cost-effective and low-cost fabrication techniques need to be explored to reduce the cost of COMs in future large-scale applications. Post-modification of COFs is an effective strategy to improve their performance in removing environmental pollutants, but the structure-activity relationship between COMs in pollutant removal still needs further study. Therefore, there remains enormous potential for COMs to be explored in the academic field.

## Author contributions

X.L.L.: investigation, writing-original draft. Y.L. and Z.S.C.: investigation. H.Y., S.H.W.: writing-review and editing. Z.W.T., X.K.W.: writing-review and editing. All authors contributed to the discussion, and gave approval to the final version of the manuscript.

## Declaration of competing interests

The authors declare no competing interest
